# Interaction of silicon-based quantum dots with gibel carp liver: oxidative and structural modifications

**DOI:** 10.1186/1556-276X-8-254

**Published:** 2013-05-29

**Authors:** Loredana Stanca, Sorina Nicoleta Petrache, Andreea Iren Serban, Andrea Cristina Staicu, Cornelia Sima, Maria Cristina Munteanu, Otilia Zărnescu, Diana Dinu, Anca Dinischiotu

**Affiliations:** 1 Department of Biochemistry and Molecular Biology, University of Bucharest, 91-95 Splaiul Independentei, Bucharest, 050095, Romania; 2 Department of Preclinical Sciences, University of Agricultural Sciences and Veterinary Medicine, 105 Splaiul Independentei, Bucharest, 050097, Romania; 3 Laser Department, National Institute of Laser, Plasma and Radiation Physics, 409 Atomistilor, Bucharest-Magurele, 077125, Romania

**Keywords:** Silicon-based quantum dots, Gibel carp, Liver, Oxidative stress, Antioxidant enzymes, Fluorescence, 68.65.Hb, 87.85.jj, 81.07.Ta

## Abstract

Quantum dots (QDs) interaction with living organisms is of central interest due to their various biological and medical applications. One of the most important mechanisms proposed for various silicon nanoparticle-mediated toxicity is oxidative stress. We investigated the basic processes of cellular damage by oxidative stress and tissue injury following QD accumulation in the gibel carp liver after intraperitoneal injection of a single dose of 2 mg/kg body weight Si/SiO_2_ QDs after 1, 3, and 7 days from their administration.

QDs gradual accumulation was highlighted by fluorescence microscopy, and subsequent histological changes in the hepatic tissue were noted. After 1 and 3 days, QD-treated fish showed an increased number of macrophage clusters and fibrosis, while hepatocyte basophilia and isolated hepatolytic microlesions were observed only after substantial QDs accumulation in the liver parenchyma, at 7 days after IP injection.

Induction of oxidative stress in fish liver was revealed by the formation of malondialdehyde and advanced oxidation protein products, as well as a decrease in protein thiol groups and reduced glutathione levels. The liver enzymatic antioxidant defense was modulated to maintain the redox status in response to the changes initiated by Si/SiO_2_ QDs. So, catalase and glutathione peroxidase activities were upregulated starting from the first day after injection, while the activity of superoxide dismutase increased only after 7 days. The oxidative damage that still occurred may impair the activity of more sensitive enzymes. A significant inhibition in glucose-6-phosphate dehydrogenase and glutathione-S-transferase activity was noted, while glutathione reductase remained unaltered.

Taking into account that the reduced glutathione level had a deep decline and the level of lipid peroxidation products remained highly increased in the time interval we studied, it appears that the liver antioxidant defense of *Carassius gibelio* does not counteract the oxidative stress induced 7 days after silicon-based QDs exposure in an efficient manner.

## Background

The extensive research of nanoparticles in connection to their various biological and medical applications has been the preamble for the development of quantum dots (QDs). These represent a heterogenous class of nanoparticles composed of a semiconductor core including group II-VI or group III-V elements encased within a shell comprised of a second semiconductor material [1]. Due to their unique optical and chemical properties, i.e., their broad absorption spectra, narrow fluorescence emission, intense fluorescence, and photo bleaching resistance [2,3], QDs were proposed as nanoprobes which were able to replace the conventional organic dyes and fluorescent proteins [4]. The use of different core material combinations and appropriate nanocrystal sizes has rendered QDs useful in biosensing [5], energy transfer [6], *in vivo* imaging [7], drug delivery [8], and diagnostic and cancer therapy applications [9].

Despite their special properties, most types of QDs have limited use in biology and medicine due to their toxicity [10]. Numerous concerns regarding the cytotoxicity of different types of QDs were presented in a recent review [11], which detailed that QD toxicity depends on a number of factors including the experimental model, concentration, exposure duration, and mode of administration.

Interestingly, efforts to reduce QD toxicity include the encapsulation in a SiO_2_ shell [7,12], with silicon-based QDs being expected to be less toxic than heavy metal-containing ones. Due to previously known benefits of silicon, like reduced elemental toxicity, its potential biodegradability to silicic acid and its abundance and low costs are adding to the promising results of recent investigations that indicate silicon use in *in vivo* imaging to be a good alternative to cadmium QDs [13,14]. Nanoporous and microparticulate forms of silicon have shown great promise in terms of compatibility and cytotoxicity [15]. Nonetheless, studies concerned with the biological and medical applications of silicon-based QDs are less numerous and still at preliminary stages [16-18].

A step towards overcoming the toxicity issue is to elucidate the *in vivo* distribution and biological effects of QDs that due to their variable characteristics must be addressed individually. It is now accepted that nude nanoparticles, including QDs, become entrapped in the cells of the reticuloendothelial system and are preferentially transported and accumulated into the liver, spleen, and also in the kidney [4,19-24]. Once localized at this levels, nanoparticles interact with the surrounding tissue and cells [25].

*In vitro* and *in vivo* studies suggest that intracellular reactive oxygen species (ROS) production is a possible mechanism for silicon-based QDs toxicity [16,26-28]. ROS are formed continuously in all living aerobic cells as a consequence of both oxidative biochemical reactions and external factors, with them being involved in the regulation of many physiological processes [29]. When the production of ROS exceeds the ability of the antioxidant system to balance them, oxidative stress occurs [30]. Because ROS are highly reactive, most cellular components are prone to oxidative damage. Consequently, lipid peroxidation, protein oxidation, reduced glutathione (GSH) depletion, and DNA single strand breaks could be initiated by ROS excess. Taken together, all these changes can ultimately lead to cellular and tissue injury and dysfunction [31].

Aquatic organisms are known for their sensitivity to oxidative stress [32]. Fish possess systems for generating as well as for protection against the adverse effects of free radicals [32,33]. Due to their dependence on oxygen availability in their environment, fish metabolism has adapted to diminish oxygen requirements. More interestingly, carp and gibel carp are capable to tolerate anoxia for periods that extend to months, depending on temperature [34]. Similarly to other aestivating animals, these fish have developed remarkable antioxidant defense mechanisms to cope with the return to normal environmental conditions [35]. The most potent antioxidant mechanisms are found particularly in the organs with high metabolic activity such as the liver, kidney, and brain [36]. Thus, the freshwater fish *Carassius gibelio* is a suitable model system to evaluate the changes induced by QDs and their putative oxidative stress related effects.

In this study, we highlighted the *in vivo* accumulation of silicon-based QDs and described the histological changes that occurred in the hepatic tissue of the gibel carp. We also focused on revealing the biochemical alterations that appeared. We evaluated the GSH concentration and the levels of oxidative stress markers such as: malondialdehyde (MDA), carbonyl derivates of proteins (CP), protein sulfhydryl groups (PSH), and advanced oxidation protein products (AOPP). Additionally, we concentrated on the activity of the antioxidant enzymes, such as superoxide dismutase (SOD), catalase (CAT), glutathione peroxidase (GPX), and glutathione-S-transferase (GST), as well as glutathione reductase (GR) and glucose 6-phosphate dehydrogenase (G6PDH) due to their key roles in antioxidant defense.

## Methods

### Chemicals

Nicotinamide adenine dinucleotide phosphate disodium salt (NADP^+^), nicotinamide adenine dinucleotide phosphate reduced tetrasodium salt (NADPH), and 1,1,3,3-tetramethoxy propane were supplied by Merck (Darmstadt, Germany). The Detect X® Glutathione Colorimetric Detection Kit was purchased from Arbor Assay (Michigan, USA), and 2,4-dinitrophenylhydrazine was from Loba-Chemie (Mumbai, India). All other reagents were purchased from Sigma (St. Louis, MO, USA), which were of analytical grade.

### Nanoparticles

The nanoparticles used in our experiment have a crystalline silicon (Si) core covered by an amorphous silicon dioxide (SiO_2_) surface. The Si/SiO_2_ nanoparticles were prepared by pulsed laser ablation technique [37]. The particles are spherical with a crystalline Si core covered with a 1- to 1.5-nm thick amorphous SiO_2_ layer. The diameter of the QDs was estimated by transmission electron microscopy image analysis. The size distribution is a lognormal function, with diameters in the range between 2 and 10 nm, with the arithmetic mean value of about 5 nm. The photoluminescent emission measured at room temperature reached maximum intensity at approximately 690 nm (approximately 1.8 eV) [38]. A suspension of nanoparticles (2 mg/mL) prepared in 0.7% NaCl was used in the current experiment.

### Animal and experimental conditions

The freshwater carp *C. gibelio* with a standard length of 13 ± 2 cm, weighing 90 ± 10 g were acquired from the Nucet Fishery Research Station, Romania. The fish were allowed to adjust to laboratory conditions for 3 weeks prior to the experiment. The fish were reared in dechlorinated tap water at a temperature of 19 ± 2°C and pH 7.4 ± 0.05, dissolved oxygen 6 ± 0.2 mg/L (constant aeration), and CaCO_3_ 175 mg/L, with a 12-h photoperiod. Fish were fed pellet food at a rate of 1% of the body weight per day. Animal maintenance and experimental procedures were in accordance with the *Guide for the Use and Care of Laboratory Animals*[39], and efforts were made to minimize animal suffering and to reduce the number of specimens used.

After the acclimatization period, the fish were randomly divided in groups of 18. Group I represented the control and consisted of fish intraperitoneally (IP) injected with 0.7% NaCl. Group II was the experimental group, and the fish were IP injected with a dose of 2 mg/kg QDs (prepared in 0.7% NaCl) per body weight. No food was supplied to the fish during the experimental period, and no obvious changes in fish body weight were recorded. After 1, 3, and 7 days from QDs injection, six fish from each group were sacrificed by trans-spinal dissection and the liver was quickly removed. Organs were immediately frozen in liquid nitrogen and stored at -80°C until biochemical analyses were performed.

### Preparation of tissue homogenates and total protein measurements

Liver was homogenized (1:10 *w*/*v*) using a Mixer Mill MM 301 homogenizer (Retsch, Haan, Germany) in ice-cold buffer (0.1 M Tris-HCl, 5 mM ethylenediaminetetraacetic acid (EDTA), pH 7.4), containing a few crystals of phenylmethylsulfonyl fluoride as protease inhibitor. The resulting homogenate was centrifuged at 8,000×*g* for 30 min, at 4°C. The supernatant was decanted, aliquoted, and stored at -80°C until needed. Protein concentration was determined using Lowry’s method with bovine serum albumin as standard [40] and was expressed as mg/mL.

### Oxidative stress markers

#### Lipid peroxidation

Lipid peroxidation was determined by measuring MDA content according to the fluorimetric method of Del Rio [41]. Briefly, 700 μL of 0.1 M HCl and 200 μL of a sample with a total protein concentration of 4 mg/mL were incubated for 20 min at room temperature. Then, 900 μL of 0.025 M thiobarbituric acid was added, and the mixture was incubated for 65 min at 37°C. Finally, 400 μL of Tris-EDTA protein extraction buffer was added. The fluorescence of MDA was recorded using a Jasco FP750 spectrofluorometer (Tokyo, Japan) with a 520/549 (excitation/emission) filter. MDA content was calculated based on a 1,1,3,3-tetramethoxy propane standard curve with concentrations up to 10 μM. The results were expressed as nanomoles of MDA per milligram of protein.

#### Protein sulfhydryl groups assay

The protein thiols were assayed using 4,4′-dithiodipyridine (DTDP) according to the method of Riener [42]. A volume of 100 μL of total protein extract was mixed with 100 μL of 20% trichloracetic acid (TCA) and thoroughly homogenized. After 10 min on ice, the samples were centrifuged at 10,000×*g* for 10 min. The pellet was rendered soluble in 20 μL 1 M NaOH and mixed with 730 μL 0.4 M Tris-HCl buffer (pH 9). Then, 20 μL of 4 mM DTDP were supplemented, and after 5-min incubation at room temperature (in the dark), the absorbance at 324 nm was measured. The concentration of PSH was quantified using a N-acetylcysteine standard curve with concentrations up to 80 μM. The values were expressed as nanomoles per milligram of protein.

#### Carbonyl derivates of proteins

CP were quantified using the reaction with 2,4-dinitrophenylhydrazine (DNPH) according to the method described by Levine [43]. The tissue extract was diluted to 500 μL to render a 0.1 mg/mL protein solution which was mixed 1:1 with 10 mM DNPH (this latter solution was prepared in 2 mM HCl). Sample blanks were prepared in a similar manner, except DNPH was excluded. Proteins were TCA-precipitated, and free DNPH was removed by washing the resulting pellets with ethanol/ethyl acetate (1:1 *v*/*v*). The pellets were rendered soluble in 600 μL 1 M NaOH and incubated for 15 min at 37°C. Sample absorbance was determined at 370 nm against its corresponding blank. CP concentration was calculated using the molar absorption coefficient of 22,000 M^-1^ cm^-1^. The results are expressed as nanomoles per milligram of protein.

#### Advanced oxidation protein products assay

The concentration of AOPP was assessed according to the method of Witko-Sarsat [44]. A sample of 200 μL total protein extract (diluted to about 0.5 mg/mL) was mixed with 10 μL 1.16 M potassium iodide and vortexed for 5 min. A volume of 20 μL of glacial acetic acid was added, and the mixture was vortexed again for 30 seconds. Sample optical density was read at 340 nm in a microplate reader. For quantification, a chloramine-T standard curve with concentrations up to 100 μM was used. The AOPP level was expressed as nanomoles per milligram of protein.

#### Antioxidant enzymes activity

SOD activity was assessed by measuring the NADP&Eta; oxidation by the superoxide radical at 340 nm [45]. This reaction sequence generates superoxide from molecular oxygen in the presence of EDTA, MnCl_2_, and mercaptoethanol. Reagent blanks were run with each set of analyzed samples, and the percent inhibition of NADPH oxidation was calculated as sample rate/blank rate × 100. One unit (U) of SOD activity was defined as the amount of enzyme that inhibited NADPH oxidation by 50% compared to the maximal oxidation rate of the reagent blank.

CAT activity was assessed following Aebi's method, which measures the decrease in absorbance at 240 nm due to H_2_O_2_ disappearance. One unit of CAT activity is the amount of enzyme that catalyzed the conversion of 1 μmole H_2_O_2_ in 1 min [46].

Total GPX activity was assayed by a method using tert-butyl hydroperoxide and reduced GSH as substrates [47]. The reduction of NADPH to NADP^+^ was recorded at 340 nm, and the concentration of NADPH was calculated using a molar extinction coefficient of 6.22 × 10^3^ M^-1^ cm^-1^. One unit of activity was defined as the amount of enzyme that catalyzes the conversion of 1 μmole of NADPH per minute under standard conditions.

GST was measured by monitoring the formation of an adduct between GSH and 1-chloro-2,4-dinitrobenzene(CDNB) at 340 nm [48]. One unit of GST activity was defined as the amount of enzyme that catalyzed the transformation of one μmole of CDNB in conjugated product per minute. The extinction coefficient 9.6 mM^-1^ cm^-1^ was used for the calculation of CDNB concentration.

The activity of GR was determined by measuring the decrease in OD at 340 nm due to NADPH consumption in a reaction medium containing the enzyme's substrate oxidized glutathione (GSSG) [49]. One unit of GR activity was calculated as the quantity of enzyme that consumed 1 μmole of NADPH per minute.

G6PDH activity was measured by the rate of the NADPH formation [50]. One unit of activity was defined as the amount of G6PDH that produces 1 μmole of NADPH per minute.

#### Reduced glutathione assay

GSH levels were determined using the Detect X® colorimetric detection kit (Sigma-Aldrich, St. Louis, MO, USA) following the manufacturer's instructions. Briefly, the tissue homogenate was deproteinized with 5% sulfosalicylic acid and analyzed for total glutathione and GSSG. GSH concentration was obtained by subtracting the GSSG level from the total glutathione. The GSSG and GSH levels were calculated and were expressed as nanomoles per milligram of protein.

#### Histology

Freshly prelevated fragments of gibel carp liver were fixed in Bouin solution or 4% paraformaldehyde in PBS, dehydrated in ethanol, cleared in toluene, and embedded in paraffin. Sections (6-μm thick) were used for hematoxylin-eosin (H&E) staining and fluorescence microscopy.

#### Fluorescent image analysis of nanoparticles distribution

After deparafination and rehydration, the slides were stained with 4,6-diamidino-2-phenylindole (DAPI) solution, mounted in PBS, and analyzed by epifluorescence microscopy using a DAPI/FITC/Texas red triple band filter set (Carl Zeiss, Oberkochen, Germany). Under ultraviolet excitation, silicon-based quantum dots appear red, and nuclei appear blue with DAPI. The photomicrographs were taken with a digital camera (AxioCam MRc 5, Carl Zeiss) driven by an Axio-Vision 4.6 software (Carl Zeiss).

#### Statistical analysis

All data presented in this paper are shown as relative values ± the relative standard deviation (RSD). The relative values were obtained by dividing the mean values registered in the experimental fish group (*n* = 6) with the mean values for the corresponding control group (*n* = 6). The differences between control and experimental groups at each time interval were analyzed by Student's *t* test and validated by confidence intervals using Quattro Pro X3 software (Corel Corporation, Mountain View, CA, USA). The results were considered significant only if the *P* value was less than 0.05, and confidence intervals of control and samples did not overlap. All biochemical assays were run in triplicate.

## Results and discussion

The applications of QDs in biological and medical area showed the tremendous potential of these nanoparticles in terms of developing new therapeutic approaches. As a result of these, it has become increasingly important to understand the biological response to their administration, considering that the main limitation in QD applications is their alleged toxicity.

### Microscopy studies

**Figure 1:**
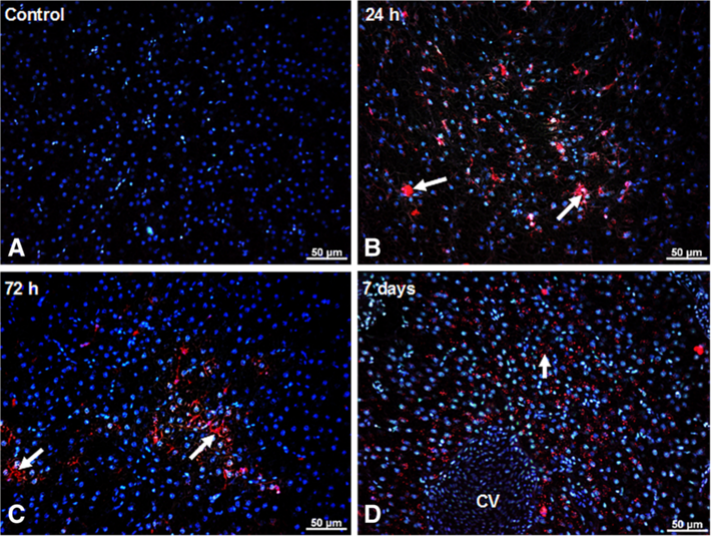
**QDs localization and accumulation in the liver of *Carassius gibelio *is highlighted by fluorescence microscopy.** When excited in UV, the DAPI-stained nuclei appear blue, while the Si/SiO_2_ QDs appear red due to their intrinsic fluorescence. (**A**) Liver tissue from control (non-injected) animals. QDs are visible in the hepatocytes at 24 h (**B**), 72 h (**C**), and 7 days (**D**) after IP injection (arrows).

Due to intrinsic photoluminescence under ultraviolet excitation, silicon-based QDs have been detected in tissue sections (Figure 1A,B,C,D). The QD characteristic red fluorescent emission was not detected for any of the control fish groups (Figure 1A). Fluorescence microscopy observations have indicated that silicon-based QDs were present and accumulated in the hepatic tissue at all time intervals (1, 3, and 7 days) (Figure 1B,C,D). The most intense accumulation was detected 7 days after IP injections, in hepatocytes around blood vessels (Figure 1D).

A histological assessment was performed to determine if silicon-based QDs accumulation cause liver damage.

**Figure 2:**
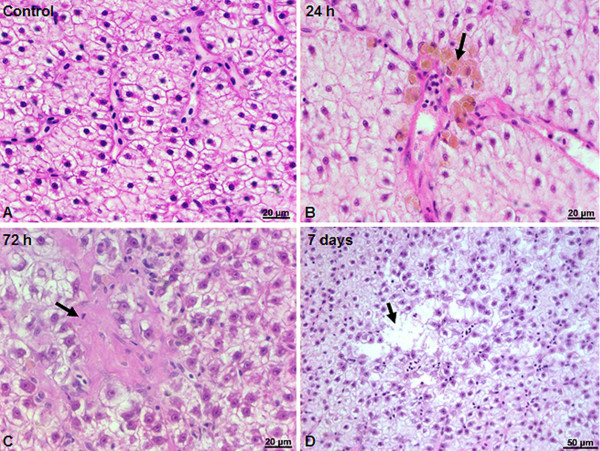
**Liver histology of *Carassius gibelio*.** (**A**) Control (non-injected) animals. (**B**) Liver histopathology 24 h after IP injection indicates accumulation of melanomacrophage centers (arrow). (**C**) Fibrosis (arrow) 72 h after IP injection. (**D**) Hepatolysis micro centers (arrow) at 7 days after IP injection. H&E staining.

 The livers of control fish showed normal histology (Figure 2A). Fish liver is composed of branching and anastomosing cords of polygonal hepatocytes, with a central, dictinctive, and hyperchromatic nucleus, with a visible nucleolus. To be more specific, extensive vacuolations are observed, a characteristic of cultured fish hepatocytes, which often become swollen with glycogen or neutral fat. In the liver of fish injected with silicon-based QDs, we observed some hystological alterations. Although functional phagocytic cells are occasionally observed in the sinusoids of healthy liver tissue, after 1 day of QDs exposure, we highlight an increased number of macrophage cluster (Figure 2B). Aggregates of macrophages are involved in recycling, sequestration, and detoxification of endogenous and exogenous compounds [51-53]. Several pathological states such as starvation [53], parasite attack [54], nutritional imbalances [55], and hemolytic anemias [53], can enhance macrophage aggregate appearance. After 3 days, the proliferation of fibrous connective tissue near sinusoids occurred, substituting liver parenchyma (Figure 2C). Hepatic fibrosis appeared, probably due to the accumulation of extracellular matrix components [56]. Oxidative stress induces fibroblast [57] and hepatic stellate cell proliferation [58] and also collagen synthesis [59]. Hepatocyte basophilia and pronounced destruction of the liver arhitecture at 7 days after IP injection were observed (Figure 2D). The cummulative effects produced by Si/SiO_2_ QDs accumulation are possibly causing a certain degree of hepatic insufficiency in gibel carp. Nonetheless, only a reduced healthy hepatic parenchyma is required to maintain normal liver function [60].

### Oxidative stress markers

The silicon quantum dots uptaken in the liver could interact with NADPH oxidase in plasma membrane, thus generating superoxide in the extracellular space [61], which would enter the cells through an anion channel [62]. Then, this anion can be transformed into hydrogen peroxide [63] which might cause a decrease in the abundance of complex III core subunit 2 and consequently a disturbance of the respiratory chain leading to ROS generation [64]. Because it is highly reactive, ROS may oxidize the most cellular compounds.

**Figure 3:**
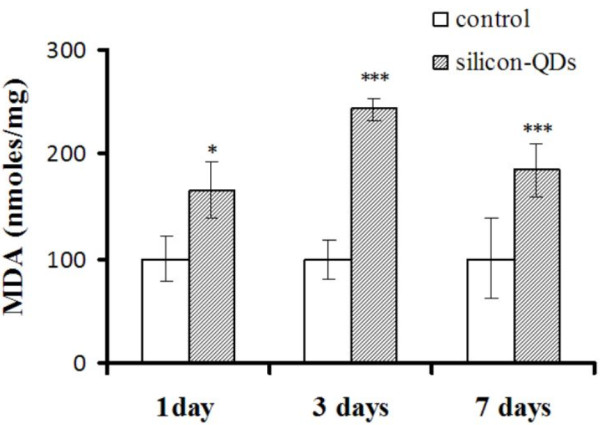
**Effects of silicon-based QDs on lipid peroxidation in *Carassius gibelio *liver.** Results are expressed as percent (%) from controls ± RSD *(n* = 6); ^*^P < 0.05; ^***^P < 0.001.

Malondialdehyde is an end product of lipid peroxidation that is extensively used as an indirect marker of oxidative stress [65]. IP injection of silicon-based QDs induced an increase of the MDA level by 66% and 143% in the liver tissue after 1 and 3 days, followed by a slight decrease after 7 days (Figure 3).

**Figure 4:**
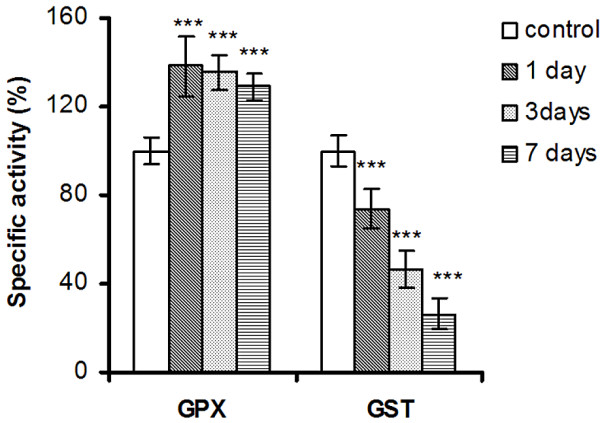
**GPX and GST specific activities in liver of *Carassius gibelio *injected with silicon-based QDs.** Results are expressed as percent from controls ± RSD (*n* = 6); ^*^P ≤ 0.05; ^**^P ≤ 0.01.

The observed MDA pattern can be explained by taking into account the various factors. Firstly, as thermoconformers, fish present acclimatory adaptations that include the enrichment of membrane lipid composition

 with polyunsaturated fatty acids (PUFA) of the *ω*-3 and/or *ω*-6 types for preserving membrane fluidity at lower temperatures. A typical reaction during ROS-induced damage is the peroxidation of unsaturated fatty acids [66]. Since the relative oxidation reaction speed generally increases with increasing unsaturation [65], fish phospholipid membranes are more sensitive to oxidative reactions by ROS than those of the mammals [67]. Hence, the highest level of MDA registered 3 days after QDs exposure might suggest strong on-going lipid peroxidation processes propagated by lipid radicals that may also affect the

 proteins (Table 1). Secondly, due to its propagative nature, lipid peroxidation of unsaturated fatty acids is less dependent on the initial level of free radicals; once initiated, it generates more reactive radicals that sustain the oxidative reaction [65]. The decreased MDA level noticed in the seventh day might be explained by the action of liver antioxidant mechanisms which are able to gradually quench the spread of lipid peroxidation that is accomplished by the activation of GPX specific activity (Figure 4). Proteins are sensitive to direct ROS attack and also to oxidative damage by lipid peroxidation products [68]. Lipid radical transfer has been demonstrated for reactive N group side chain aminoacids tryptophan, arginine, histidine, and lysine. Tyrosine and methionine degradation by oxidizing lipids has also been demonstrated [69]. Due to their reactivity, lipid peroxidation end products such asmalondialdehyde or other lipid-derived aldehydes do not accumulate and they form Schiff bases in the reaction of carbonyl groups with the amino groups of proteins.

The effects of the silicon-based QDs exposure on protein oxidation in the liver tissue of *C. gibelio* are summarized in Table 1. In our experiment, a sudden AOPP increase by 83.5% is highlighted starting with the first day postexposure. The presence of infiltrating macrophages in the hepatic parenchyma, also noted at this early time point (Figure 2B), can account for the increased AOPP level. AOPP are formed subsequent to

**Table 1:** Protein oxidative alterations

**Time (days)**	**AOPP**		**PSH**		**CP**	
	**Control**	**Exposed**	**Control**	**Exposed**	**Control**	**Exposed**
1	100 ± 13	183.5 ± 17^**^	100 ± 3	87.2 ± 10^*^	100 ± 13	98.4 ± 11
3	100 ± 16	191.5 ± 21^**^	100 ± 9	65 ± 5^**^	100 ± 12	102.3 ± 10
7	100 ± 10	208.9 ± 14^**^	100 ± 6	51 ± 13^**^	100 ± 9	90.9 ± 17

Carbonyl derivates of proteins (CP), advanced oxidation protein products (AOPP), and protein thiol groups (PSH) in liver of fish after 1, 3, and 7 days of silicon-based QDs exposure. Results are presented expressed as percent from controls ± RSD (*n* = 6); ^*^P < 0.05; ^**^P < 0.01.

 neutrophil myeloperoxidase activation, by the action of hypochlorite that selectively attacks proteins, aiming primarily at the lysine, tryptophan, cysteine, and methionine residues.

Current literature supports the role of protein thiol groups as prime ROS targets. In fact, PSH can scavenge 50% to 75% of intracellular generated ROS, suffering reversible or irreversible oxidations during this process [68]. Our data showed that PSH were reduced in the liver of fish IP injected with Si/SiO_2_ QDs (Table 1). After 1 day, the PSH level diminished by about 13% while, for longer periods, the decrease was amplified, i.e., it was reduced by 35% after 3 days and by 49% after 7 days. The continuous decrease of PSH over the 7-day period may imply that sufficient PSHs were available to be oxidized and thus explain the protection from more severe protein oxidative damage, such as carbonylation. Our current results indicated that protein carbonylation is not a characteristic alteration in silicon-based QD-induced oxidative stress in the liver since protein carbonyls maintained at a basal level (Table 1). Our previous results indicated a decrease in PSH content in the kidney of *C. gibelio*[70], while in white muscle tissue, this parameter remained unchanged after QDs administration [71]. These differences are probably due to the QDs *in vivo* distribution, since the liver is a main target

 of QDs accumulation and the kidney is involved in the nanoparticles clearance, whereas white muscle accumulated QDs to a lesser extent due to its poor vascularization.

### Antioxidant defense system

**Figure 5:**
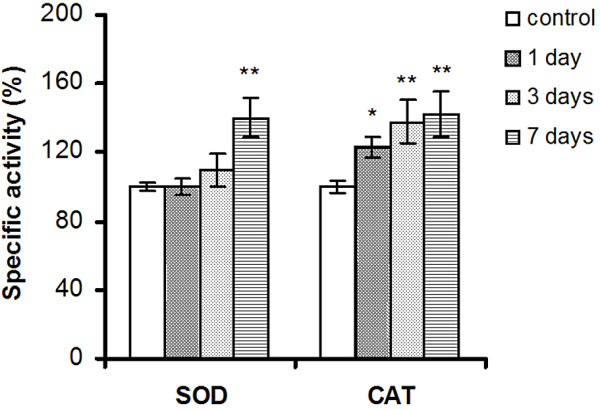
**The effect of silicon-based QDs on the SOD and CAT activities in *Carassius gibelio* liver.** Results are expressed as percent from controls ± RSD (*n* = 6); ^***^P ≤ 0.001.

The liver enzymatic antioxidant defense is modulated in response to the redox status changes initiated by Si/SiO_2_ QDs. Figure 5 shows the different responses of SOD and CAT to silicon-based QDs accumulation in the liver of *C. gibelio*. These differences may be explained on the account of their functions. SOD activity increased by 40.1% after 7 days of QDs administration, whereas no significant changes in the activity of this enzyme were noticed in the first 3 days. SOD eliminates the free radical superoxide by converting it to hydrogen peroxide, which, in turn, is cleared by CAT. Several pathways are involved in the production of superoxide in normal cells and tissues such as xanthine oxidase, the mitochondrial electron transport system enzymes, NAD(P)H oxidase, etc. [72]. The interaction of silicon QDs with these pathways after substantial tissue accumulation may account for the increased superoxide radical input a week after QDs exposure.

Our data show distinct changes in CAT activity, which is elevated at every time interval studied, with the most notable increase of 42% measured in the seventh day

 after Si-based QDs administration. The progressive induction of CAT would indicate the emergence of an increasing source of hydrogen peroxide during a 7-day period after QDs IP injection. It is well established that H_2_O_2_ is produced through two-electron reduction of O_2_ by cytochrome P-450, D-amino acid oxidase, acetyl coenzyme A oxidase, or uric acid oxidase [73]. Additionally, Kupffer cells, which are fixed to the endothelial cells lining the hepatic sinusoids have a great capacity to endocytose exogenous particles (including QDs) and secrete large amounts of ROS [74]. Since the amount of QDs in the liver accumulates gradually and is at a maximum after 7 days, we suggest that the substrate for CAT must be generated by the QDs directly or indirectly. It is possible that the early activation of CAT may be due to an increased production of H_2_O_2_ by a mechanism different from ·O_2_^-^ dismutation. Indeed, the fact that H_2_O_2_ generation may be central to silica nanoparticle toxicity has recently been deduced, since catalase treatment decreases the nanotoxic effects of SiO_2_ nanoparticles [75].

The activity of GPX increased after 1 day of exposure by 38% and remained approximately at this level in the next days (Figure 4). GPX works in concert with CAT to scavenge the endogenous hydrogen peroxide, but GPX has much higher affinity for H_2_O_2_ than CAT suggesting that this enzyme acts *in vivo* at low H_2_O_2_ concentrations whereas CAT is activated at high substrate concentrations [76]. The early activation of liver GPX and the persistence of almost the same level of activity throughout the experiment may be due to other functions of the enzyme, like lipid radical detoxification.

The GSTs are a group of multifunctional proteins, which play a central role in detoxification of hydroperoxides, by conjugation with GSH [35]. An accentuated decrease in the levels of GST activity was observed post-QDs treatment (Figure 4). At low GSH concentrations, cytosolic GST is inhibited by the binding of alpha, beta-unsaturated carbonyl derivatives to specific cysteine residues of the enzyme [77]. Such unsaturated carbonyl derivates are formed by non-enzymatic Hock cleavage of susceptible phospholipid molecules that contain PUFA acyl chains [78].

**Figure 6:**
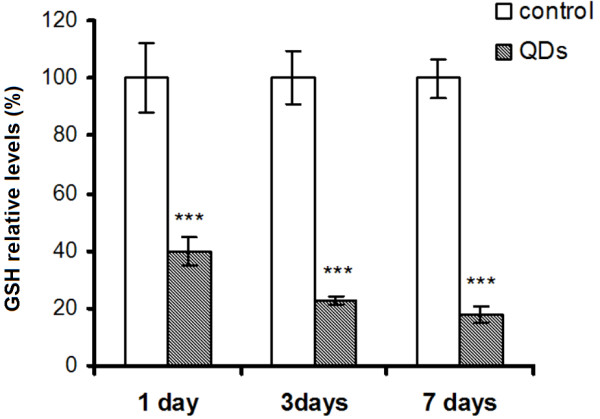
**GSH concentration in the liver of *Carassius gibelio *after silicon-based QDs administration.** Results are expressed as percent from controls ± RSD (*n* = 6); ^***^P ≤ 0.001.

**Figure 7:**
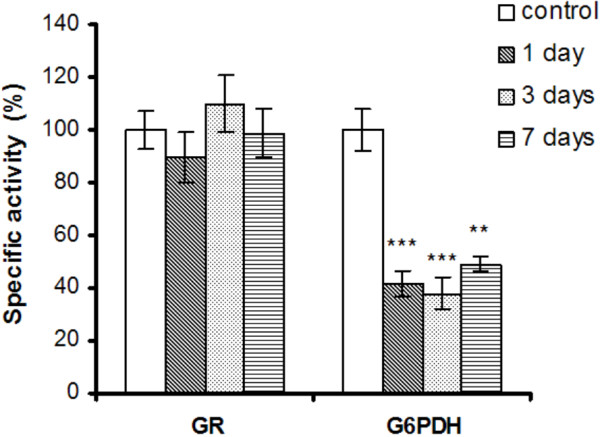
**GR and G6PD specific activities in liver of *Carassius gibelio *injected with silicon-based QDs exposure.** Results are expressed as percent from controls ± RSD (*n* = 6); ^**^P ≤ 0.01, ^***^P ≤ 0.001.

A central role in managing the cellular redox status is held by GSH. This tripeptide has a dual role serving both as a free radical scavenger by itself as well as a substrate for GPX and GST. The GSH concentration decreased by 60%, 78%, and 83% after 1, 3, and 7 days of QDs treatment, compared to the corresponding controls (Figure 6). This depletion cannot be explained by the adaptative upregulation of GPX activity only. Also, we have to take into consideration the contribution of GSH conjugation with prooxidants and the hindrance of GSH reservoir replenishment due to the GR unchanged activity (Figure 7). A decrease of intracellular GSH level was

 also reported in RAW 267.7 cells treated with silica nanoparticles [27]. Hepatic GSH depletion by 20% has been shown to impair the cell's defense against ROS and is known to cause liver injury [79].

G6PDH catalyzes the first reaction of pentose phosphate pathway and generates NADPH involved in reductive biosynthesis and antioxidant defense. It has been demonstrated that G6PDH ablation has deleterious metabolic consequences, including the impairment of hydrogen peroxide detoxification [80]. After 1 day of exposure, the activity of G6PDH decreased by about 50% and remained reduced throughout the experiment (Figure 7). Being a rate-limiting enzyme in the NADPH synthesis pathway, a decrease in the NADPH/NADP^+^ ratio probably occurred. The reduced activity of G6PDH can be explained by the decrease of protein thiols, which may consequently impair many enzymes [81]. Indeed,

 cysteine along with histidine and arginine residues was shown to be essential for G6PDH activity [82].

The liver GR is essential for the recycling of GSSG to GSH, and it requires NADPH as co-substrate. NADPH depletion may impede the upregulation of GR in order to counteract GSH oxidation. This observation is supported by other studies that showed no significant alteration in the level of GR in human epithelial cells in the presence of pure silica nanoparticles [17].

The results reported in the literature concerning QDs toxicity appear very divergent, and careful consideration must be given to the differences in chemical composition, size, and dosage as well as the experimental model chosen in the respective studies. Our data are in agreement with the previous reports which reported the ROS formation as a primary mechanism for toxicity of silicon nanoparticles [16,26-28,75]. However, the data available in regard to oxidative stress marker and antioxidant systems exposed to silicon QDs are limited. The results of this study provide new but strong evidences of the direct effects on proteins and lipids as targets of oxidative stress induced by silicon-based QDs. The induction of some antioxidants enzyme could explain the lesser toxicity of these QDs. The information on cellular state offered by this study may be essential to nanoparticle areas, helping to understand the extent to which silicon QDs perturb the biological system.

## Conclusions

The results reported here make a valuable contribution to the further understanding of the *in vivo* toxicity of Si/SiO_2_ QDs on short and medium term, especially by outlining the mechanisms involved in generating their deleterious effects. Oxidative stress induced in fish liver by silicon-based QDs following their accumulation is highlighted by the formation of MDA and AOPP and the decrease of PSH and GSH. The modulation of the major antioxidant enzymes suggests a response mounted towards maintaining the redox status, since both GPX and CAT (with a later activation of SOD) are upregulated. The oxidative damage that still occurred impaired the activity of more sensitive enzymes, like GST, GR, and G6PGH, which in turn further contributed to hinder the recovery. These biochemical alterations became more intense as QDs liver accumulation gradually increased. The most extensive histological alterations, including fibrosis and the formation of microfoci of hepatolysis were also observed after significant QD accumulation, at 3 and 7 days, respectively, from their IP injection. A longer period of time from Si/SiO_2_ exposure may be needed in order to overcome their harmful effects. We also believe that lower doses of Si/SiO_2_ QDs should be relatively biocompatible, and careful adjustment of QD dosage may open the way for their successful use in various *in vivo* imaging applications.

## Competing interests

The authors declare that they have no competing interests.

## Authors' contributions

LS and SNP carried out the biochemical studies. ACS carried out the animal experiment and contributed in the integration of histological studies with the biochemical results. MCM participated in the design of the research. Histological determination and interpretation were performed by OZ. DD analyzed the experimental results and drafted the manuscript. AD conceived of the study and participated in its design and coordination. AIS performed some of the experiments. CS planed the experimental design. All authors read and approved the final manuscript.
